# Enantioselective additions of copper acetylides to cyclic iminium and oxocarbenium ions

**DOI:** 10.3762/bjoc.11.290

**Published:** 2015-12-22

**Authors:** Jixin Liu, Srimoyee Dasgupta, Mary P Watson

**Affiliations:** 1 Department of Chemistry and Biochemistry, University of Delaware, Newark, DE 19716, USA

**Keywords:** catalysis, copper, enantioselectivity, iminium ion, oxocarbenium ion

## Abstract

The development of enantioselective, copper-catalyzed alkynylations of cyclic iminium and oxocarbenium ions is reviewed. The use of chiral copper-based catalysts has enabled high yields and enantioselectivites in the formation of nitrogen- and oxygen-containing heterocycles with α-stereogenic centers. This review highlights both the accomplishments and the future work needed in this important area.

## Introduction

Nitrogen and oxygen heterocycles with α-stereogenic centers represent important classes of biologicially active compounds [1–7]. Enantioselective addition of chiral nucleophiles to imines, iminium ions, carbonyls, or oxocarbenium ions provides efficient access to these scaffolds. In particular, exceptional progress has been made in the addition of chiral metal acetylides. Historically, these reactions required stoichiometric amounts of both metal and chiral ligand [8], but catalytic variants are now available with a variety of metal-based catalysts [9–15]. Among these, chiral copper catalysts have been used with remarkable success in the alkynylation of cyclic iminium ion and oxocarbenium ion intermediates. This review will focus on the development of these enantioselective, copper-catalyzed alkynylations, highlighting both the accomplishments and the future work needed in this important area.

**Figure 1:**
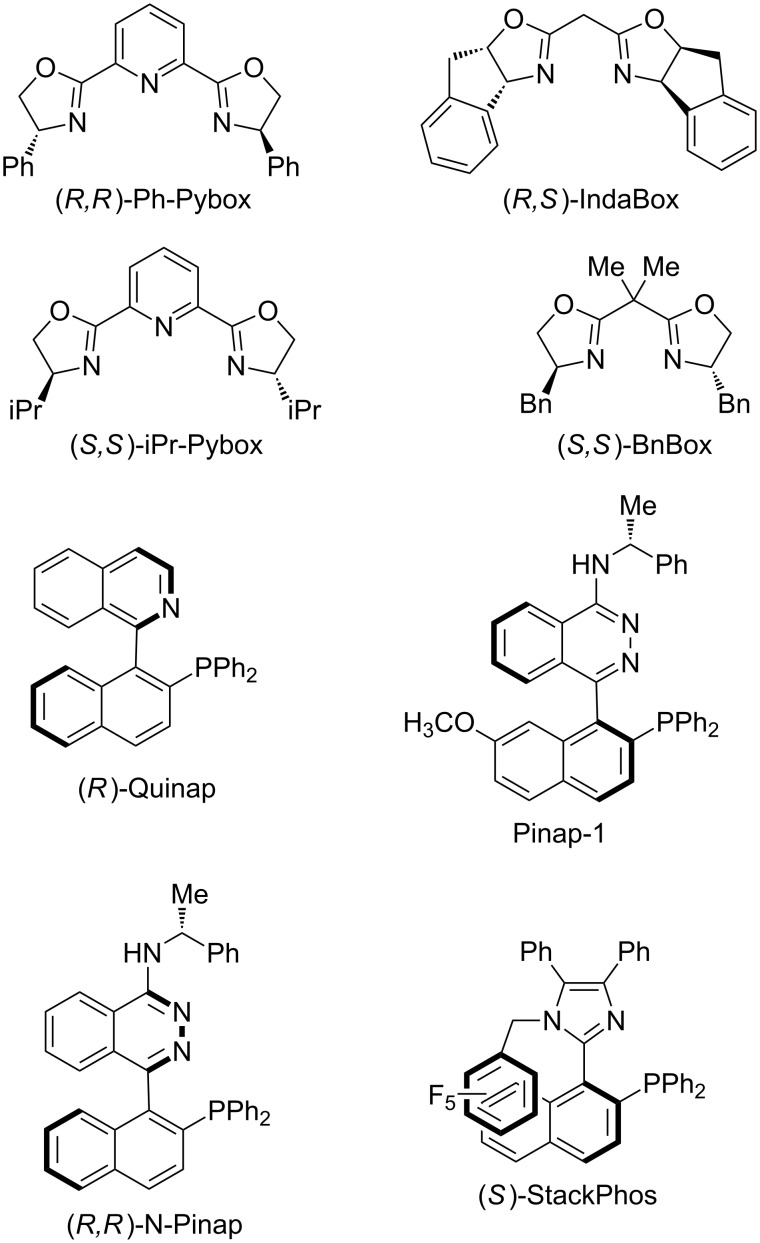
Chiral ligands utilized in copper-catalyzed alkynylations of cyclic iminium and oxocarbenium ions.

Throughout the discussion below, it is clear that there are privileged ligand architectures of these copper-catalyzed alkynylations. High enantioselectivities have been achieved with pyridine bis(oxazoline) (Pybox), bis(oxazoline) (Box), and Quinap-type ligands (Figure 1).

## Review

### Additions to iminium ions

**Scheme 1:**
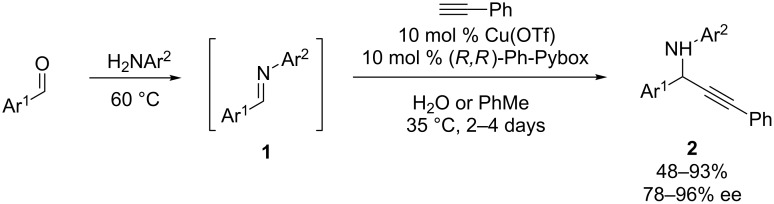
Li’s alkynylation of acyclic *N*-arylimines.

**Scheme 2:**
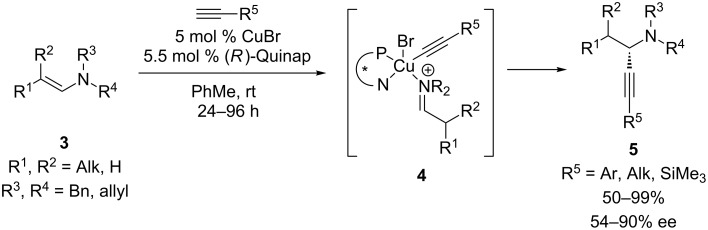
Knochel’s alkynylation of acyclic *N*-alkylenamines.

Although this review will focus on enantioselective additions to cyclic electrophiles, it is worth noting that the first enantioselective additions of chiral copper acetylides to imines or iminium ions utilized acyclic imine substrates. In 2002, Li and co-workers reported enantioselective alkynylations of *N*-aryl aldimines formed in situ from benzaldehydes and anilines (Scheme 1) [16–17]. This reaction employs a CuOTf/Ph-Pybox catalyst system to achieve generally high yields and ee’s of propargylic amines **2**. Notably, this reaction can be carried out in H_2_O, as well as PhMe. Nearly simultaneously, Knochel’s group reported an enantioselective CuBr/Quinap-catalyzed alkynylation to deliver propargylic amines with alkyl substitution at the stereocenter (Scheme 2) [18–20]. In this reaction, proton transfer from the terminal alkyne to the enamine simultaneously generates the copper acetylide and iminium ion, which are proposed to both bind to the chiral copper catalyst (see **4**). A broad scope in the acetylene was observed, with arylalkynes resulting in the highest ee’s. To our knowledge, these were the first enantioselective, metal-catalyzed additions of terminal alkynes to imines or iminium ions, and set the stage for subsequent development of enantioselective alkynylations of cyclic iminium ion substrates [21]. As discussed below, the catalyst systems identified in these reactions have largely informed those used for enantioselective alkynylations of cyclic electrophiles.

**Scheme 3:**
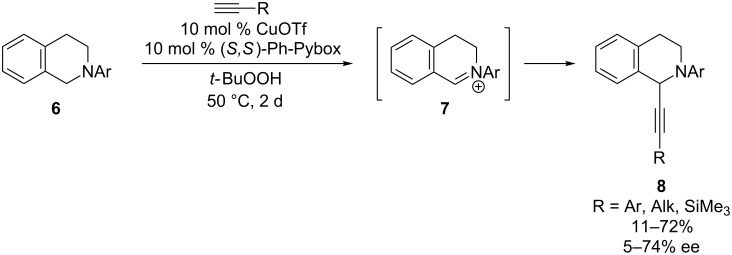
Li’s CDC of tetrahydroisoquinolines and alkynes.

The first enantioselective, copper-catalyzed alkynylation of a cyclic iminium ion was reported by Li’s research group in 2004 [22]. Building on their development of a cross-dehydrogenative coupling (CDC) reaction between benzylic amines and alkynes to deliver racemic products [23], the Li group developed a CuOTf/Ph-Pybox catalyst system that enables alkynylation of tetrahydroisoquinolines in moderate to good yields and enantioselectivies (Scheme 3). A particularly powerful aspect of this chemistry is that a stable tetrahydroisoquinoline **6** can be utilized as the substrate. Oxidation of the tetrahydroisoquinoline then results in formation of iminium ion **7** in situ. As in Knochel’s reaction above, the highest yields and ee’s were observed with arylacetylenes.

**Scheme 4:**
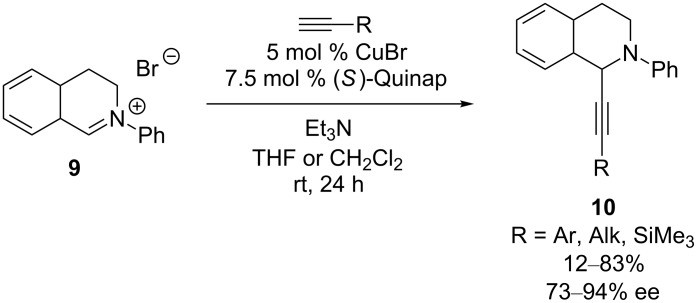
Li’s alkynylation of *N*-aryldihydroisoquinolinium ions.

In subsequent studies of this reaction, Li investigated the intermediacy of isoquinolinium ion **9**, and found that improved yields and ee’s can be achieved using this substrate and a CuBr/Quinap catalyst, despite the fact that Quinap had proven inferior to Ph-Pybox in the CDC reaction (Scheme 4) [24]. With this new catalyst and electrophile, the catalyst loading, reaction temperature, and reaction time could be reduced. Addition of alkynes with aryl, alkyl and trimethylsilyl substituents were successful, with the highest enantioselectivity observed when (trimethylsilyl)acetylene was used (94% ee).

**Scheme 5:**
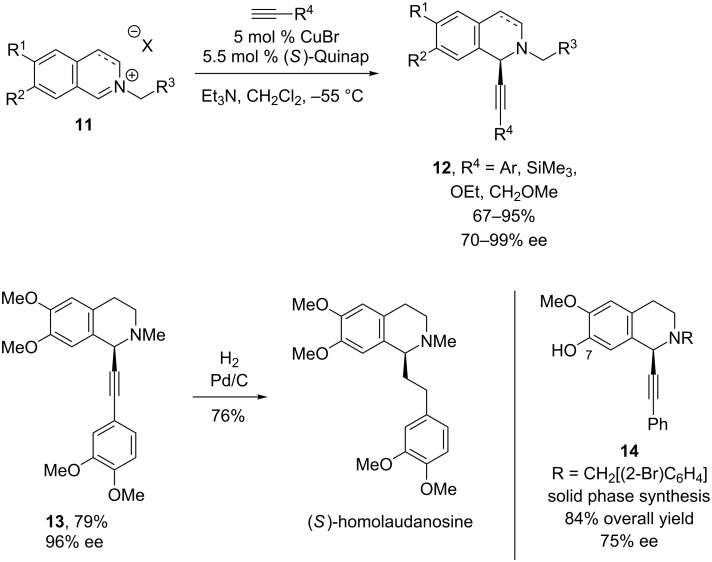
Schreiber’s alkynylation of *N*-alkylisoquinolinium ions.

Just before Li’s report of the alkynlation of *N*-arylisoquinolinium ions, Taylor and Schreiber reported a CuBr/Quinap-catalyzed alkynylation of *N*-alkylisoquinolinium ions (Scheme 5) [25]. Similar to Li’s alkynylation of *N*-arylisoquinolinium ions, alkynes with various substituents can be used successfully, and the highest ee’s were observed when (trimethylsilyl)acetylene was used (99% ee). Most of the iminium ion substrates were dihydroisoquinolinium ions, but alkynylation of the aromatic isoquinolinium ion was also achieved in 67% yield and 83% ee at a higher reaction temperature (−20 °C). The authors elegantly showed the potential of this reaction in two examples. First, they reduced alkyne **13** to deliver (*S*)-homolaudanosine, a natural product from an alkaloid family with neurologic activity, in high yield and enantiopurity. They also demonstrated that this alkynylation is amenable to solid phase synthesis; alkyne **14** was prepared by alkynylation of an isoquinolinium ion linked to a polystyrene bead through the C7 hydroxy group.

**Scheme 6:**
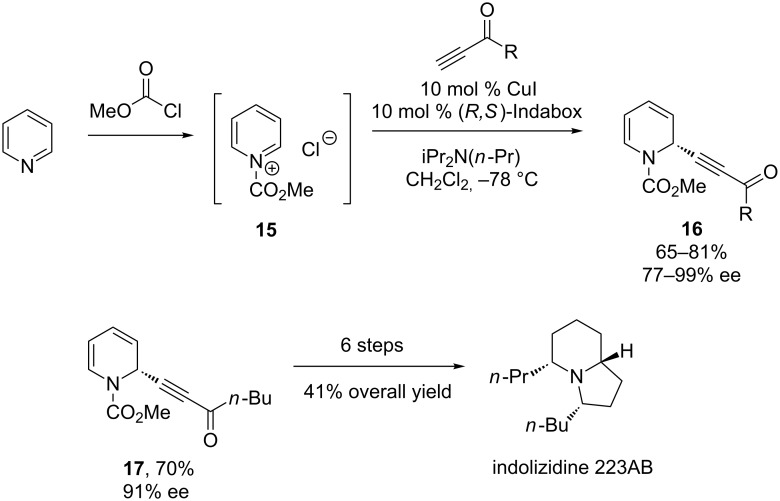
Ma’s alkynylation of pyridium ions.

In 2007, Ma and co-workers reported the first enantioselective, copper-catalyzed alkynylation of a pyridinium ion (Scheme 6) [26]. This reaction employs an *N*-acylpyridinium ion generated in situ and a CuI/Indabox catalyst. Ma found that the identity of base and solvent also affect the enantioselectivity, with iPr_2_N(*n*-Pr) and CH_2_Cl_2_ proving best. Notably, only 1,2-addition was observed. With respect to the alkyne, activated terminal acetylenes, such as ynones and propriolates, are best for this reaction. Unactivated alkynes give products in reasonable yields (63–77%), but poor enantioselectivities (1–11% ee). However, the use of activated acetylenes provides a functional group handle for elaboration, which the authors demonstrate in the preparation of indolizidine 223AB.

**Scheme 7:**
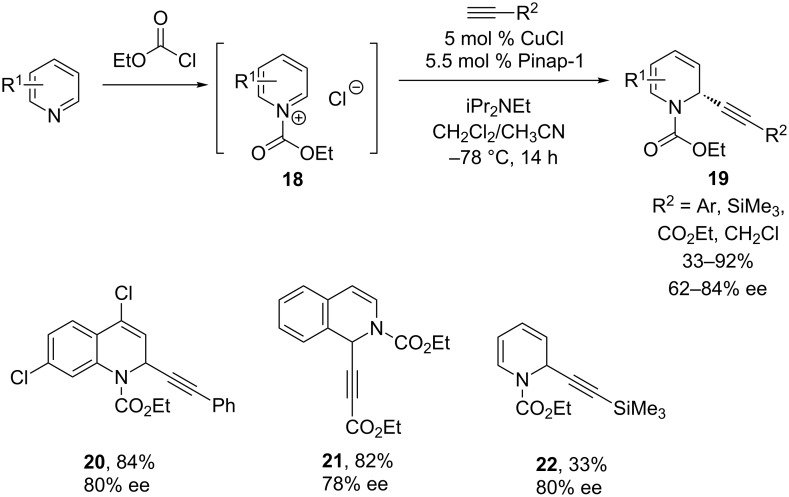
Arndtsen’s alkynylation of cyclic iminium ions.

Building on their initial discovery of a non-asymmetric, copper-catalyzed, three-component coupling of pyridine, benzoyl chloride and phenylacetylene [27], the Arndtsen group tackled the challenge of developing a catalyst for the alkynylation of cyclic *N*-acyliminium ions with unactivated alkynes [28]. Using the reaction of *N*-acylquinoline and phenylacetylene as their model, they observed lower ee’s with Pybox and Box ligands, and only 49% ee with Quinap. However, investigation of novel Pinap ligands led to notably higher ee’s (Scheme 7) [29]. These reaction conditions enable alkynylation of quinoline (**20**), isoquinoline (**21**), and pyridine (**22**) substrates, albeit in lower yields with pyridines.

**Scheme 8:**
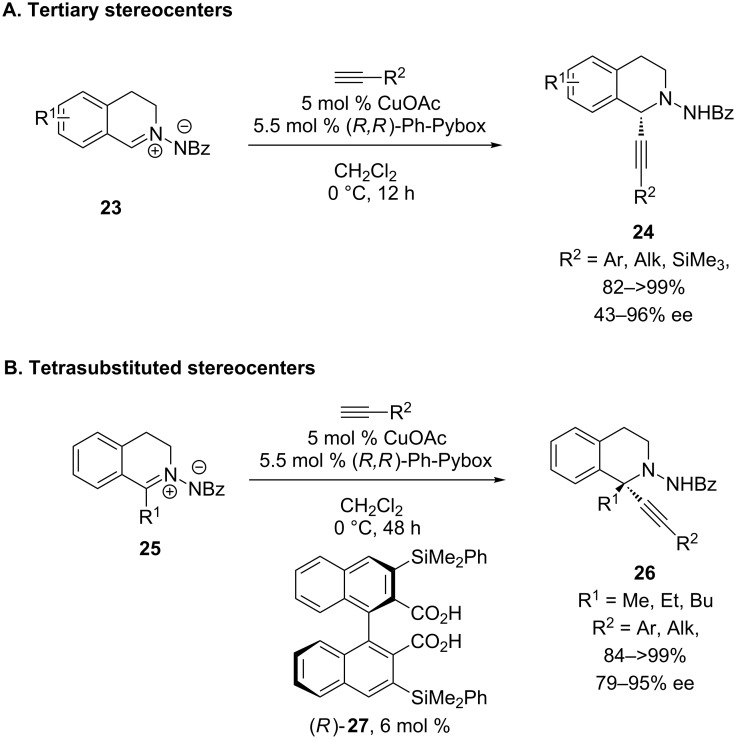
Maruoka’s alkynylation of azomethine imines.

In 2011, Maruoka and co-workers investigated the use of isoquinolinium ions protected as azomethine imines [30]. The use of a CuOAc/Ph-Pybox catalyst enables the addition of a wide variety of alkynes to form isoquinolines with tertiary stereocenters in high yields and ee’s (Scheme 8A). Although lower ee’s were observed with *o*-tolylacetylene (43% ee) and 1-heptyne (75% ee), all other alkynes resulted in ≥85% ee. Even more impressive, the authors discovered conditions for a highly enantioselective alkynylation to form tetrasubstituted stereocenters. With 1-alkylisoquinolinium ions, high ee’s could not be achieved using only a CuOAc/Ph-Pybox catalyst. Postulating that the acetate of CuOAc may facilitate proton transfer from the alkyne to the azomethine imine, a necessary step to form both the cationic iminium and the copper acetylide, Maruoka investigated the use of a chiral acid co-catalyst. With the addition of chiral Brønsted acid co-catalyst **27**, high yields and good to excellent levels of enantioselectivity were achieved in the addition of both aryl and aliphatic alkynes (Scheme 8B). The azomethine amine products **24** and **26** can be deprotected using SmI_2_. To our knowledge, this report is the first and only example to date of alkynylation of an imine or iminium ion to form a chiral tetrasubstituted center with high ee.

**Scheme 9:**
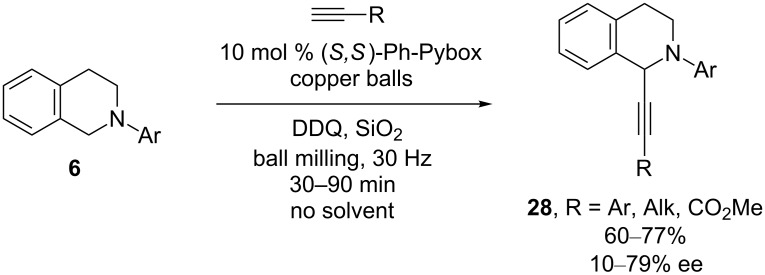
Su’s CDC of tetrahydroisoquinolines and alkynes under ball milling conditions.

Enantioselective, copper-catalyzed alkynylations have also been accomplished under solvent-free conditions. In 2013, Su and co-workers established that the CDC reaction of *N*-aryltetrahydroisoquinolines and alkynes can be accomplished under high-speed ball-milling conditions with copper balls (Scheme 9) [31]. Under these solvent-free reaction conditions, isoquinolines **28** were formed more quickly than the in-solvent reactions previously reported, but the ee’s are lower.

**Scheme 10:**
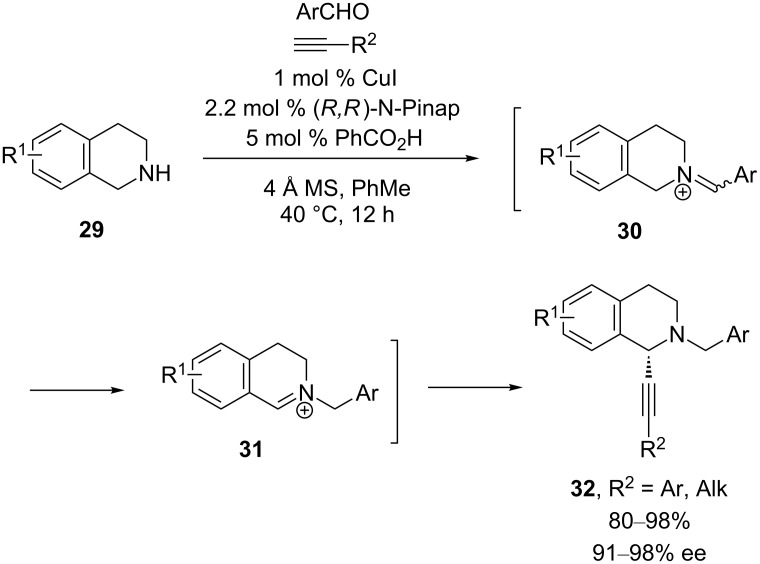
Ma’s A^3^-coupling.

Recently, a new entry to isoquinolinium ions has been established. In their studies of the three-component coupling of aldehydes, amines, and alkynes (A^3^ reaction), the Ma group serendipitiously discovered that isomerization of exocyclic iminium ion **30** results in the formation of endocyclic iminium ion **31** (Scheme 10) [32]. Subsequent alkynylation was accomplished using a CuI/N-Pinap catalyst to give *N*-benzylisoquinolines in exceptional yields and enantioselectivities. Notably, catalytic benzoic acid is necessary to achieve high yields. The authors hypothesize that this acid additive facilitates the iminium ion isomerization.

**Scheme 11:**
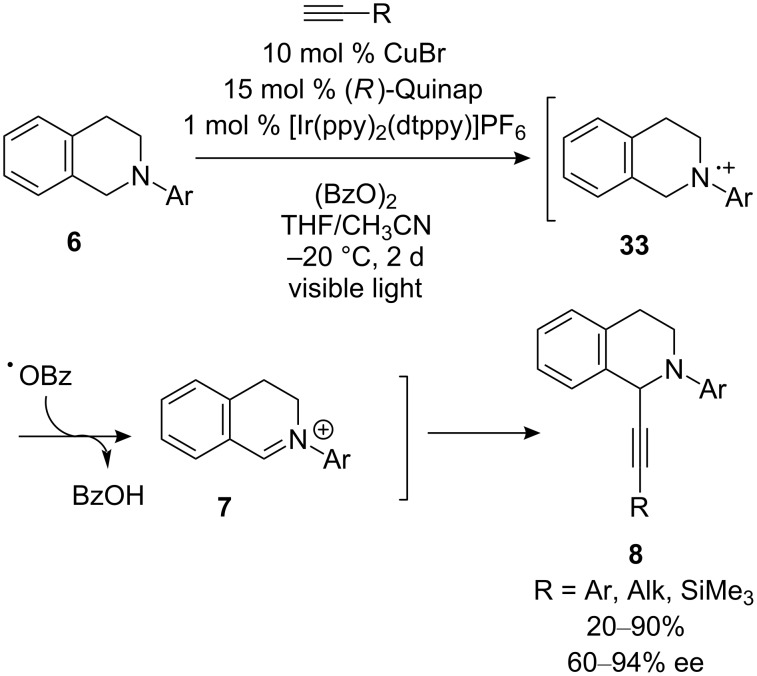
Li’s CDC reaction using photoredox catalysis.

This year, several examples of improved conditions for CDC reactions have been reported. With the emergence of photoredox catalysis as a powerful technique for organic synthesis, Li’s research group has discovered improved conditions for the CDC reaction of *N*-aryltetrahydroisoquinolines with alkynes (Scheme 11) [33]. By using an iridium-based photoredox catalyst in combination with benzoyl peroxide, iminium ion **7** is formed in situ. This strategy enables reduction of the reaction temperature, ultimately enabling higher enantioselectivities. With respect to the scope of alkynes, high ee’s were observed with both aryl- and alkylacetylenes, but lower yields were seen with alkylacetylenes. In addition, (trimethylsilyl)acetylene can be used but in lower yield and ee (40%, 60% ee).

**Scheme 12:**
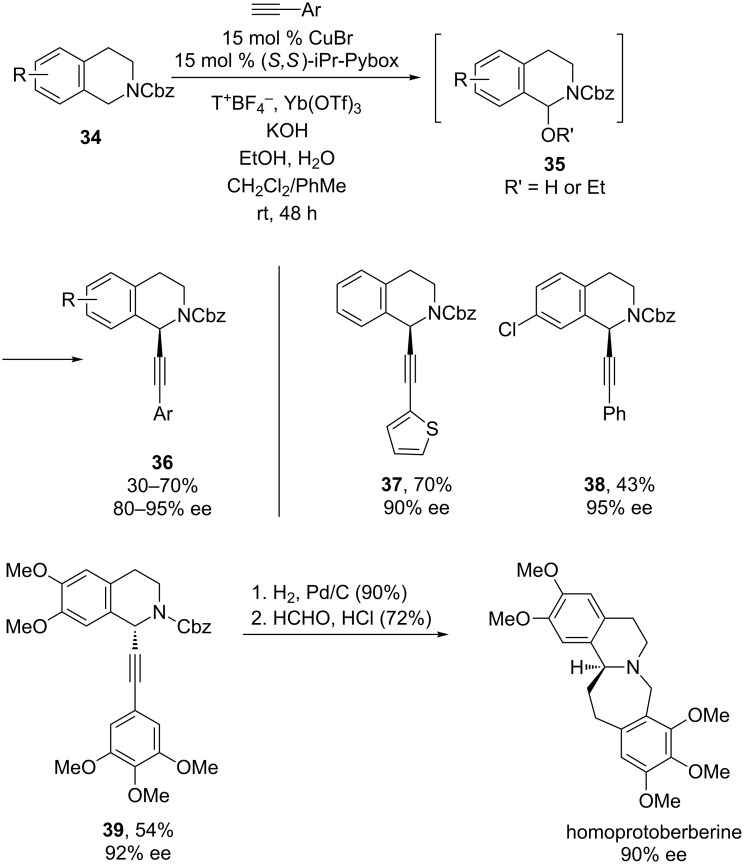
Liu’s CDC reaction of *N*-carbamoyltetrahydroisoquinolines. T^+^BF_4_^–^ = 2,2,6,6-tetramethylpiperidine *N*-oxide tetrafluoroborate.

Also in 2015, Liu and co-workers have discovered conditions that enable the use of *N*-carbamoyltetrahydroisoquinolines in CDC reactions with alkynes (Scheme 12) [34]. These reactions utilize a CuBr/iPr-Pybox catalyst with 2,2,6,6-tetramethylpiperidine *N*-oxide as the oxidant. Liu’s mechanistic experiments indicate that the reaction likely proceeds via hemiaminal **35**, formed via oxidation to an iminium ion and subsequent trapping by either EtOH or H_2_O. With respect to the substrate scope, addition of arylalkynes proceeds in high yields and ee’s, including those with some heteroaryl groups (**37**). Enynes can also be added, but result in lower yields and ee’s, as do octyne and methyl propriolate. A variety of substituents are tolerated on the isoquinoline, including halides, which provides a handle for further manipulation. Furthermore, the Cbz group can be easily removed via hydrogenation, as demonstrated in Liu’s efficient synthesis of homoprotoberberine.

**Scheme 13:**
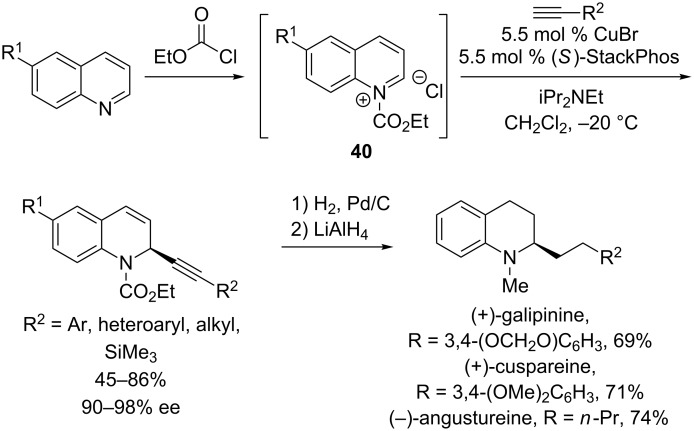
Aponick’s alkynylation of *N*-carbomoylquinolinium ions using StackPhos as ligand.

Very recently, Aponick’s group reported the use of a new P,N ligand, StackPhos, for enantioselective alkynylations of quinolinium ions [35]. They hypothesized that their imidazole-based P,N ligand would provide a different bite angle than Quinap or Pinap ligands and thus enable higher ee’s than previously obtained in the alkynylation of these challenging aromatic iminium ions [28]. Indeed, the CuBr/StackPhos catalyst provides generally high yields and exceptional levels of enantioselectivity in the alkynylation of *N*-carbamoylquinolinium ions **40** (Scheme 13). A broad scope was observed with the alkyne partner; additions of alkynes with aryl, heteroaryl, alkyl, and trimethylsilyl substituents result in 90–98% ee. The synthetic utility of these products, as well as their absolute configuration, was demonstrated by their reduction to the natural products (+)-galipinine, (+)-cuspareine, and (−)-angustureine.

### Additions to oxocarbenium ions

**Scheme 14:**
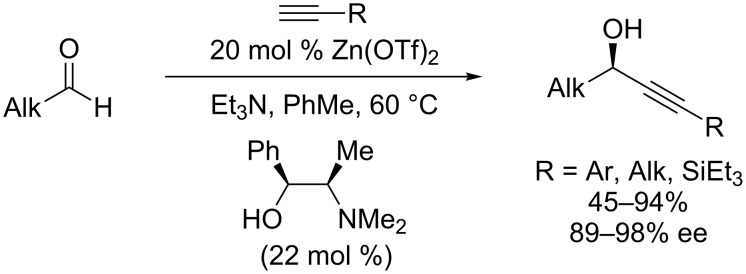
Carreira’s enantioselective, catalytic alkynylation of aldehydes.

Similar to the development of enantioselective, metal-catalyzed alkynylations of iminium ions, enantioselective alkynylations of cyclic oxocarbenium ions were preceded by enantioselective alkynylations of acyclic aldehyde and ketone substrates [36–54]. In particular, seminal reports by the Carreira lab demonstrated that the use of stoichiometric metal acetylides was not required to achieve addition to aldehydes; catalytic Zn(OTf)_2_ and *N*-methylephedrine in combination with Et_3_N enabled in situ formation of chiral zinc acetylides, which underwent addition to aldehydes in good yields and high enantioselectivities (Scheme 14) [38]. Since this report, a variety of metal catalysts, including copper-based catalysts [36–37,50–52], have been employed in enantioselective alkynylations of aldehydes and ketones [36–54]. However, in contrast to the efforts made in enantioselective, metal-catalyzed additions to cyclic iminium ions, much less attention has been focused on analogous reactions of cyclic oxocarbenium ions. Given the precedent in alkynylations of aldehydes and ketones, as well as the alkynylations of iminium ions discussed above, we have pursued the development of enantioselective, metal-catalyzed alkynylation of cyclic oxocarbenium ion intermediates. In the course of these studies, we have found that copper-based catalysts are uniquely effective in promoting these alkynylations in good yields and ee’s.

**Scheme 15:**
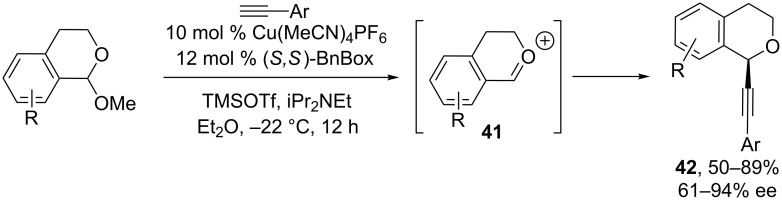
Watson’s alkynylation of isochroman oxocarbenium ions.

In 2011, we reported the first example of enantioselective alkynylation of a cyclic oxocarbenium ion intermediate (Scheme 15) [55]. Isochroman oxocarbenium ion **41** was formed in situ via Lewis acid-mediated ionization of a racemic acetal precursor. By using a Cu(MeCN)_4_PF_6_/BnBox catalyst, moderate to high yields and enantioselectivities were achieved in the addition of arylalkynes. Both yields and ee’s drop with vinyl- or alkylalkynes.

**Scheme 16:**
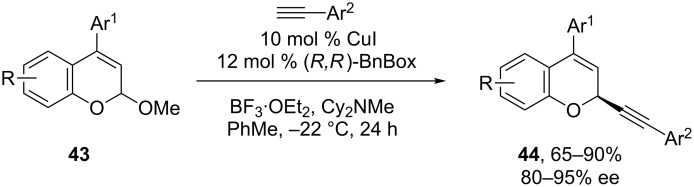
Watson’s alkynylation of chromene oxocarbenium ions.

Recently, we have demonstrated that enantioselective, copper-catalyzed alkynylations of oxocarbenium ions derived from chromene acetals **43** can also be achieved in high yields and ee’s (Scheme 16) [56]. In this case, the reaction concentration has a significant impact on the enantioselectivity. We also observed that the alkynylation of chromene acetals with electron-donating substituents proceeds in higher ee’s than less electron-rich substrates, suggesting that more stable oxocarbenium ions result in more selective reactions, potentially because they lead to later enantiodetermining transition states.

**Scheme 17:**
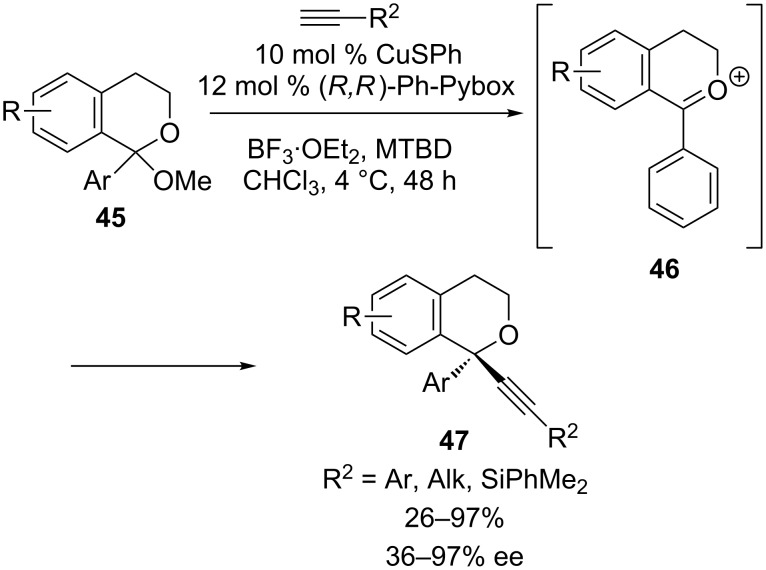
Watson’s alkynylation to set diaryl tetrasubstituted stereocenters.

We have also discovered a CuSPh/Ph-Pybox catalyst that enables the formation of diaryl, tetrasubstituted stereocenters via an enantioselective alkynylation reaction (Scheme 17) [57]. Remarkably, by tethering one aryl group to the oxygen atom, the two faces of oxocarbenium ion **46** can be distinguished by the catalyst. In general, excellent yields and enantioselectivities were observed, although lower yields and ee’s are seen with certain aliphatic alkynes. The addition of (dimethylphenylsilyl)acetylene proceeds in 53% yield and 81% ee, providing a silylalkyne that can be readily deprotected in quantitative yield for further elaboration. Notably, comparison of the ee of ligand vs ee of product revealed a significant positive nonlinear effect, indicting that catalyst aggregation occurs under these reaction conditions [58]. It is unclear at this point, whether these catalyst aggregates are on or off the catalytic cycle.

## Conclusion

As described above, highly enantioselective, copper-catalyzed alkynylations of cyclic iminium and oxocarbenium ion intermediates have been achieved. α-Chiral piperidines, quinolines, isoquinolines, and benzopyrans are accessible via these reactions. The utility of these alkyne-substituted products has been demonstrated via elaboration to biologically active natural products. As evidenced by the most recent reports, particularly for iminium ions, progress is on-going to determine stable precursors to the requisite iminium ion intermediates and to identify readily removed protecting groups.

Given the potential of enantioselective, copper-catalyzed alkynylations to deliver important scaffolds, significant effort is still required to develop this class of reactions. In particular, all cyclic iminium and oxocarbenium ions utilized to date have been limited to those that form stabilized cationic intermediates (benzylic or aromatic). With few exceptions, the vast majority lack β-hydrogens, so competitive elimination reactions are not possible. Conditions to enable the use of non-stabilized iminium and oxocarbenium ions with β-hydrogens would represent an exceptional advance in this field and allow access to a wide variety of useful compounds.

In addition, little is understood about how these chiral copper catalysts provide high levels of enantioselectivity. This lack is likely due to the multiple possibilities for copper acetylide structures (monomer, dimer, dicopper acetylide, etc.) [18,59–65]. Careful mechanistic studies to elucidate the structures of chiral copper acetylides and to provide stereochemical rationale for the enantioselectivities of these reactions is needed to enable further development of catalysts in this important area.
